# Editorial Brevities

**Published:** 1887-03

**Authors:** 


### EDITORIALS AND MISCELLANEOUS.

### EDITORIAL BREVITIES.

DR. M. M. EVANS requests us to notify his friends that he has changed his Post-Office from Faulkner Gap to Tyro P. O., Arkansas.

PRACTICE is the name of a new medical journal published in Richmond, Va., and edited by J. F. Winn, M. D. It is neatly gotten out, and edited with ability. We welcome it to our exchange list, and wish the genial and intelligent editor great success in his worthy enterprise.

BICYCLES.—According to the Mirror of American Sports there are upward of 250 doctors in the United States who make use of the bicycle as a means of health and conveyance. Dr. Richardson, the great London physician, is an enthusiastic wheelman.

